# Help Us Help You: Engaging Emergency Physicians to Identify Organizational Strategies to Reduce Burnout

**DOI:** 10.5811/westjem.2020.49180

**Published:** 2021-04-08

**Authors:** Joshua J. Baugh, Ali S. Raja, James K. Takayesu

**Affiliations:** Harvard Medical School, Massachusetts General Hospital, Department of Emergency Medicine, Boston, Massachusetts

## Abstract

**Introduction:**

Burnout is a major threat to patient care quality and physician career longevity in emergency medicine. We sought to develop and implement a quality improvement process to engage emergency department (ED) faculty in identifying sources of burnout and generating interventions targeted at improving the work environment.

**Methods:**

In this prospective interventional study conducted at a large, urban, academic medical center, we surveyed a 60-person faculty group using the Professional Fulfilment Index (PFI), as well as burnout-relevant questions from the American Medical Association’s Mini-Z survey and the Maslach-Leiter framework for organizational burnout, in order to identify organizational sources of burnout. We assessed the relationship between burnout scores and responses to the Maslach-Leiter framework using univariate regression analysis. In a two-hour facilitated session, we shared survey results and led the group in a process using the six Maslach-Leiter domains to develop a rank-ordered list of interventions to reduce burnout in each domain.

**Results:**

In total, 47 of 60 faculty (78.3%) completed the survey and 45 faculty (75%) attended the discussion session. Of the 47 survey respondents, 14 (30%) met criteria for moderate to severe burnout. The respondents’ answers to the Maslach-Leiter organizational burnout domain questions were significantly correlated with their burnout scores (P <0.001). Session attendees generated 31 potential interventions for process improvement, which were analyzed and thematically organized. Common intervention themes included reducing documentation burden, receiving more positive feedback on patient care, improving ease of obtaining consults, decreasing ED crowding, and increasing intrafaculty social connection. Interventions were subsequently reviewed and scored based on relative importance and feasibility to create a departmental action plan for process improvement.

**Conclusion:**

Using the Maslach-Leiter organizational burnout framework, in conjunction with a facilitated solution-oriented faculty discussion, led to the creation of a departmental agenda focused on organizational solutions for augmenting professional fulfillment and reducing burnout. We propose that this process can be used by healthcare organizations to engage physicians and others in efforts to improve their work experiences, which in turn is likely also to support the provision of higher quality of care.

## INTRODUCTION

Physician burnout is particularly common in emergency medicine (EM), with deleterious effects on both career longevity and patient care quality.^1^–^3^ The majority of burnout research across medicine has focused on solutions that require individual action, such as mindfulness training, yoga, and personal reflection.^4^ While studies suggest that approaches focused on individuals can be moderately helpful for alleviating burnout,^5^,^6^ other work, both in medicine and across industries, has found that the majority of burnout is potentiated by organizational factors rather than individual ones.^7^,^8^ Multiple studies in medicine have now demonstrated that an organizational approach to mitigating burnout can be effective.^9^ However, an optimal process for diagnosing and addressing organizational causes of burnout has not yet been established.

In decades-long work across many industries, Christina Maslach and Michael Leiter identified six organizational domains intimately related to workforce burnout.^7^ These six domains are as follows: workload; reward; control; fairness; community; and value congruence. They also found that these same domains – when optimized – could foster engagement, the antithesis of burnout. This six-domain framework has recently been applied specifically to EM, producing an array of potential interventions for promoting engagement over burnout in emergency physicians (EP).^10^ Maslach and Leiter’s work also suggests that engaging workers in the organizational improvement process can itself help to alleviate burnout by 1) identifying the right target issues affecting staff most frequently, and 2) empowering individuals to change their work environment in ways that improve the experience of work.

We sought to create an approach for engaging our own EM faculty in such a process to identify areas for departmental improvement. We employed the Maslach-Leiter framework to organize a facilitated, solution-oriented discussion incorporating departmental measures of burnout and professional fulfillment to elicit ideas from the group on how to improve the department across the six organizational domains. We describe a process using survey data combined with a two-hour faculty discussion session to frame burnout from an organizational perspective with the goal of developing interventions to address sources of burnout by creating a comprehensive departmental process-improvement plan.

## METHODS

### Study Design and Setting

We conducted this prospective interventional study at a large, urban, academic medical center with a Level I trauma center and STEMI center designations. We surveyed a 60-person faculty group of board-certified/board-eligible EPs. This project was deemed quality improvement by our organization and exempted from formal review by our institutional review board.

### Survey Creation and Distribution

We used the full, validated 16-item Professional Fulfillment Index (PFI) to measure individuals’ burnout and sense of fulfillment from their clinical work.^11^ Response options consisted of a five-point Likert scale, and questions were grouped to form three PFI scales: professional fulfillment; work exhaustion; and interpersonal disengagement. In addition, we added specific questions from the American Medical Association Mini-Z survey^12^ to measure the influence of specific workplace issues known or suspected to be related to negative experiences at work (eg, documentation burden) not explicitly assessed by the PFI. Last, we asked respondents to rate how the department was performing in each of the six Maslach-Leiter domains of organizational burnout (Table 1), using questions that we developed. The survey also contained an open-response section in which participants could identify issues they found to be the most frustrating aspects of their work experience.

The survey was distributed through Qualtrics (Qualtrics XM, Provo, UT) to each faculty member two weeks prior to the discussion session. To encourage participation, we informed participants that the data from the survey would inform the discussion session. Three reminder emails were sent after the initial survey distribution.

### Survey Analysis

We calculated an overall burnout score, as well as individual PFI scores for depersonalization, emotional exhaustion, and professional fulfillment, for each participant based on the criteria developed and validated for the PFI.^11^ The relationship of burnout scores to responses to each of the six Maslach-Leiter domain questions was calculated using univariate regression analysis for each individual question, using an alpha error threshold of 0.05 for statistical significance. We analyzed the open responses for recurring themes.

### The Discussion Session

Prior to the beginning of the discussion session, we distributed one-page sheets with the six Maslach-Leiter domains listed, along with pens, to be used for note-taking later in the session. (See below.) The session was divided into five phases (Table 2).

In phase 1, we reviewed the previous departmental initiatives undertaken since the last retreat session two years prior addressing burnout, including creation of a mini-sabbatical for long-tenured faculty, sharing of positive clinical feedback, additional ED coverage for surge times of day, addition of a lactation room, and individualizing schedule preferences. We presented overall rates of burnout, as well as de-identified scores in the three PFI subcategories. In phase 2, we introduced the Maslach-Leiter framework and then presented the departmental ratings in each of the six Maslach-Leiter domains. For each domain, we presented the overall score, highlighted prior efforts for improvement in the domain, and reviewed select de-identified comments from the survey relating to each domain. The goal of this phase was to create a foundation for solution-oriented discussion.

For phase 3, we asked individuals to use the note-taking sheets to write down ideas for process improvement in each of the six domains that could build upon previous improvements or address new unmet needs. Phases 1, 2, and 3 took approximately 45 minutes in total.

In phase 4, individuals shared their ideas with a small group (randomly selected groups of 5–6 faculty based on seating location) with the goal of generating 1–2 departmental interventions that would be most desirable to improve clinical work and academic productivity. Groups were asked to come to a consensus on their most important improvements for both clinical and non-clinical work. During phase 5, each group reported out on their choices of most important clinical and non-clinical improvements to the general audience. A master list was generated from this report-out, prioritized by number of mentions across the groups followed by consensus agreement. At the conclusion of the session, the one-page sheets with individual ideas for improvements in each domain were also collected for further review to ensure that all ideas were captured.

### After the Session

We analyzed the individual worksheets for content and overlapping ideas and themes. The worksheets provided a very rich data set for identifying opportunities for promoting engagement and fulfillment not included in the consensus-generated list from the report-out. The first author (JB) then reviewed the free-text comments and generated a collection of themes using modified grounded theory. The senior author (JKT) then used the theme structure identified by the first author to review the free-text comments, and no new themes were identified. Agreement on themes, as well as frequency counts of themes, was reached by both authors. The intervention themes were then rank-ordered in priority based on these frequency counts.

The prioritized list of interventions from both the report-out consensus and the individual worksheet dataset was presented to departmental leadership to assess the feasibility of implementation. A feasibility-importance chart was created, with feasibility based on cost and current operational constraints on one axis, and importance as determined by frequency of mention by faculty on the other axis. Items that were below average in both feasibility and importance were removed from the list. All other ideas remained in consideration, with highest priority given to items that ranked above average in both importance and feasibility. The items were then organized into a wellness agenda for the coming year.

## RESULTS

### Faculty Participation

Of the 60 faculty members, 47 (78%) completed the survey and 45 attended the retreat session.

### Overall and Scale-specific Burnout

Among 47 faculty survey respondents, 14 (30%) met criteria for burnout, 22 (46%) met criteria for emotional exhaustion, and 14 (30%) met criteria for depersonalization. A total of 27 (57%) met criteria for low professional fulfillment.

### Relationship of Burnout Scores to Maslach-Leiter Domains

Participants’ ratings of our department in the six Maslach-Leiter domains were highly associated with their burnout scores. Ratings of the department in five of the six individual domains were significantly associated with burnout score (Table 3). Individuals’ aggregate ratings of the six domains were also significantly associated with their burnout scores.

### Burnout Intervention Ideas

A total of 31 distinct ideas for interventions to mitigate burnout and improve faculty engagement were generated by participants during the group session. These were qualitatively analyzed (JB and JKT) by domain and organized into the 15 most commonly cited interventions (Table 4).

## DISCUSSION

Burnout is a commonly recognized issue in clinical practice across all medical specialties but is particularly prevalent in EM.^4^ We sought to understand the relationship between physician burnout and the six organizational domains identified by Maslach and Leiter within an EM context. We then aimed to use these domains as a framework to drive a solution-oriented process to identify distinct targets for improving the experience of work and professional fulfillment in our emergency department, with the specific goal of reducing burnout.

Prior research suggests that actively engaging physicians in the process of organizational improvement is important for achieving reductions in burnout.^13^ In particular, improving quality in areas of concern specifically identified by physicians has been shown to decrease burnout.^14^ Our process demonstrates an easily implementable method for beginning this type of engagement, as three-quarters of our EM faculty participated in a pre-session survey followed by a faculty retreat to generate potential department-specific strategies to address sources of burnout in clinical and academic work. The process we designed was clinician-centered, with groups building on individual ideas to develop consensus around potential interventions. These intervention ideas were then shared with and assessed by departmental leadership, resulting in an agenda for change that would be both feasible and impactful.

The Maslach-Leiter domains provided a framework for organizing the discussion of burnout interventions with concrete categories both for assessing our physicians’ work experience and developing solutions for improving that experience. It is worth noting that nearly all of the intervention ideas generated focused on improving the experience of work, rather than adding extra resources for life outside of work (eg, funding gym memberships or grocery services). This finding aligns with prior research suggesting that while still relatively rare in the medical literature, burnout interventions focused on organizational change tend to be more effective than those aimed at individual mental health.^15^ Other studies have shown that allowing physicians to spend more time on meaningful work activities can decrease burnout and that environments which promote patient satisfaction and better patient outcomes also lead to less physician burnout.^16^,^17^ Most of the interventions identified by our faculty were focused on ways of improving the efficiency of patient care and increasing research productivity, reinforcing the idea that making it easier for physicians to perform their jobs effectively can improve fulfillment and potentially reduce burnout.

Faculty ratings of the department within the six Maslach-Leiter domains were associated significantly with their burnout scores, suggesting that their experiences in these domains were related to burnout, as the framework’s underlying theory would predict. Of course, it is also possible that the causal direction was reversed – faculty experiencing burnout may have been more likely to rate departmental domains lower because of their exhaustion, depersonalization, and lack of personal accomplishment. Our process was not designed to assess for causality, but this would be a relevant question for future study. It is also unclear why “community” scores were not associated with burnout, although it may be because there was less variance in response to this question than any of the others; scores on this measure were uniformly high, potentially creating a ceiling effect.

Previous studies have suggested that effective organizational leadership decreases burnout and that practice environments identified as both “patient-friendly” and “physician-friendly” have lower burnout rates.^18^,^19^ Our process provided a way for department leaders to engage staff in identifying areas of focus for promoting better experiences and outcomes for patients. Importantly, the resulting interventions derived from the process were often “win-win,” ie, solutions were not only relevant to addressing faculty burnout but were also tightly linked to improvements in operational efficiency and care quality. We suspect other departments too will find that areas identified by staff as important for preventing burnout may also highlight opportunities for process improvement.

## LIMITATIONS

This article describes our experience with a process executed once with a single faculty group of physicians. While we believe the process is generalizable, there may be challenges in other settings that we did not encounter. Our sample for assessing the relationship between the Maslach-Leiter domains and burnout scores was small, and this study cannot be used as a validation of the questions we developed. Further work in this area is warranted. We were also unable to assess whether the process we designed, and the resulting solutions, will truly alleviate burnout and promote engagement; this will only be apparent over time.

## CONCLUSION

We developed an approach to engaging emergency physicians in developing solutions for burnout using the Maslach-Leiter organizational framework. We believe this approach can help other departments engage with physicians to improve their experience of work, with potential positive effects on both career longevity and quality of care.

## Figures and Tables

**Table 1:** The assessment statements developed for each of the six Maslach-Leiter domains. Faculty were asked to rate each of these statements on a 5-point Likert scale, from 1 = strongly disagree to 5 = strongly agree.

Maslach-Leiter domain statements (rated 1–5 from strongly disagree to strongly agree)
My workload in this job is manageable. The rewards I derive from my work are commensurate with my effort. I have sufficient autonomy and control in my work. Our workplace is fair and transparent. We have a strong sense of community in our department. Our workplace allows us to fulfill the values endorsed by our department.

**Table 2:** Phases of the two-hour facilitated discussion session on burnout, organized by activity.

Phase 1: Review of prior departmental efforts and presentation of overall burnout data.
Phase 2: Introduction of Maslach-Leiter framework and presentation of data regarding departmental performance in each domain, with examples and illustrative quotes.
Phase 3: Individual idea generation for solutions in each of the six domains.
Phase 4: Small group discussion of most important clinical and non-clinical interventions.
Phase 5: Small group report-out of intervention ideas, with generation of a final list of improvements, followed by group discussion regarding prioritization.

**Table 3:** Association of Maslach-Leiter categories with burnout scores, using univariate regression. Negative coefficients reflect that higher (better) scores in a given domain were associated with lower burnout (N = 47).

Maslach-Leiter domain	Coefficient	P-value
Workload	−0.37	<0.001
Reward	−0.27	0.005
Control	−0.48	<0.001
Fairness	−0.22	0.048
Community	0.03	0.84
Value congruence	−0.31	0.004
Average of all categories	−0.6	<0.001

**Table 4:** Most commonly mentioned interventions identified by faculty for reducing burnout, organized by Maslach-Leiter category, with total number of times mentioned in parentheses.

Category	Recommendations most commonly cited by faculty (# of times cited)
Workload	Reduce documentation burden (21) Increase administrative support for research activities (13) Augment staffing when volumes are too high (10)
Reward	Increase positive patient stories and positive feedback (12) Increase public recognition of excellence by faculty (6) Provide compensation for more activities not currently compensated (6)
Control	Improve ease and speed of consults and admissions (20) Create a method for providing feedback to other departments (5)
Fairness	Improve ability to customize schedule and work fewer night shifts (7) Improve compensation and recognition for valued non-clinical work (6)
Community	Increase frequency of social events (15) Create centralized office locations to promote socializing (7)
Value congruence	Provide less patient care in hallways and chairs (10) Reduce boarding and emergency department crowding (8) Alleviate burnout of other role groups in the emergency department (8)
